# The prophylactic effect of date palm (*Phoenix dactylifera* L.) fruit extract on testicular toxicity induced by formaldehyde: An experimental study

**DOI:** 10.18502/ijrm.v13i4.6890

**Published:** 2020-04-30

**Authors:** Mahdieh Zare, Tahereh Haghpanah, Majid Asadi Shekari, Seyed Hassan Eftekhar-Vaghefi

**Affiliations:** ^1^Department of Anatomical Sciences, School of Medicine, Kerman University of Medical Sciences, Kerman, Iran.; ^2^Health Policy Research Center, School of Medicine, Shiraz University of Medical Sciences, Shiraz, Iran.; ^3^Neuroscience Research Center, Institute of Neuropharmacology, Kerman University of Medical Sciences, Kerman, Iran.; ^4^Department of Anatomy, Kerman Branch, Islamic Azad University, Kerman, Iran.

**Keywords:** Formaldehyde, Date fruit, Testis, Toxicity, Sperm, Testosterone.

## Abstract

**Background:**

Formaldehyde (FA) is one of the most widely used materials in industries and in sciences. Prolonged contact with FA might have harmful effects on fertility due to the increase in the reactive oxygen species level. On the other hand, date palm (*Phoenix Dactilifera L.*) fruit extract (DPFE) contains a high concentration of natural antioxidants that could scavenge free radicals. **Objective:** The aim was to investigate the prophylactic effects of DPFE, with strong antioxidant properties, on FA-induced testicular toxicity in male mice.

**Materials and Methods:**

Thirty-two adult NMRI male mice with a weight range of 25-35 gr (9-10 wk old) were randomly divided into four groups: control group (distilled water, orally for 35 days), FA group (FA; 0.25 mg/kg intraperitoneally (i.p.) for 20 days), treatment group (Date (DT) + FA; DPFE, 4 mg/kg for 35 days followed by FA administration, 0.25 mg/kg, i.p., for 20 days), date fruit extract group (DT; DPFE, 4 mg/kg, orally for 35 days). After this, blood was collected and left epididymis and testis tissues were isolated to evaluate the sperm parameters and histological examination, respectively.

**Results:**

The FA administration increased the sperm morphological anomalies and reduced the sperm count, viability and motility, and also testosterone compared to the control group (p ≤ 0.001). In addition, histological studies of the testes showed that FA causes changes in the testis seminiferous tubules such as destruction of germinal epithelium and vacuolization of the tubules. The DPFE consumption before FA administration could partially ameliorate the reduced testosterone, sperm, and testicular parameters due to FA.

**Conclusion:**

The DPFE use might have discount effects on FA-induced testicular toxicity.

## 1. Introduction

Formaldehyde (FA) is one member of aldehyde family that exists in homebred air, cigarette smoke, and the polluted atmosphere of the city. It is used in histology, anatomy, pathology, and industrial production as well as agricultural industry (1, 2). Despite its widespread use, various studies have reported its harmful effects on different tissues of the body such as male reproductive system (3, 4). The negative changes of testicular and sperm parameters after exposure to FA have been reported. The harmful effects of FA on various tissues of the body such as testicular tissue could be due to excess production of reactive oxygen species and also deficiency in endogenous antioxidant systems (5).

Antioxidants may protect cells via scavenger of free radicals and therefore maybe able to decrease the effects of FA exposure on the reproductive system. Furthermore, several studies have showed that natural foods with high concentration of antioxidants could defend testes versus FA-induced stress oxidative (5, 6).

Date palm (*Phoenix dactylifera*) fruit, is one of the plants that has long been emphasized in traditional medicine for the improvement of fertility potential. This plant is an important source of food in Asia, the Middle East, and African countries, and it grows in the tropical regions of Iran, including Bam city in Kerman. The aqueous extract of *P. dactylifera* fruit contains carbohydrates and natural antioxidants such as vitamins and phenolic compounds including flavonoids, ferulic acid, p-coumaric, and anthocyanins (7). It has been shown that the use of *P. dactylifera* seed oil (8), pollen (9), and fruit (10) can improve sperm and testicular parameters as welll as the antioxidant capacity (11). The protective effect of *P. dactylifera* extract on the reproductive system has been reported after exposure to various toxins such as amirtaz (12), cadmium (13), dichloroacetic acid (14), and atrazine (10). It has been suggested that this protective effect against several toxins could be related to the antioxidant compounds present in date extract (10).

Considering several reports that demonstrated the negative effect of FA exposure on male reproductive system due to oxidative stress, the consumption of plants containing valuable natural antioxiants can be a suitable and cost-effective alternative to chemical drugs for the protection of fertility potential. Date palm fruit extract (DPFE) with high concentration of anioxidant compounds have the potential to protect the reproductive system from oxidants. Until now, the potential prophylaxic effects of the DPFE consumption on the FA-induced reproductive system were not investigated, in this study, we examine the possible protective effect of the administration of DPFE for 35 days before exposure to FA on reproductive system in male mice.

## 2. Materials and Methods

All used reagents in present study were purchased from Merck, Germany unless otherwise stated.

### Animals 

In this experimental study, 32 adult male NMRI mice with a weight range of 25-35 gr (9-10 wk old) were used. The mice were maintained in standard conditions of animal housing; 12/12 hr light/dark cycle, 25 ± 1°C, and free access to water and balanced diet ad libitum.

### Plant material

Fresh *P. dactylifera *fruit was collected from Bam city of Kerman province (southeast of Iran). A voucher specimen (accession number: KF-1638) was deposited at the Herbarium of Herbal and Traditional Medicines Research Center, Kerman University of Medical Sciences, Kerman, Iran.

### Preparation of date fruit aqueous extract

After separating the fruit core, 100 gr of fruit was immersed in 1000 milliliters (mL) of distilled water for 48 hr. Then, it was mixed in a mixer and centrifuged at 4°C with 4000 rpm for 20 min. After centrifugation, the upper part was stored at 4°C until use for gavage.

### Experimental protocol

The mice were randomly assigned to one control and three treatment groups. The control group received distilled water via gavage for 35 days. The animals in date fruit extract group (DT) received aqueous extract of date fruit via gavage at a dose of 4 mg/kg (15) for 35 days. The mice in FA group (FA) received FA intraperitoneally (i.p.) with a dose of 0.25 mg/kg (16) for 20 days. In pre-treatment group (DT + FA), animals received date fruit extract for 30 days followed by FA administration at a dose of 0.25 mg/kg intraperitoneally for 20 days every day.

### Blood sample collection

First, mice were anesthetized i.p. by an administration of ketamine (80 mg/kg body weight) /xylazine (5 mg/kg body weight). Blood was collected from the left ventricle of heart and centrifuged at 3000 rpm for 30 min. The serum was removed and kept in -20°C until use (17). The level of testosterone was measured by radioimmunoassay, using the Coat-A-Count RIA kit (Diagnostic Products Corporation, Los Angeles, California, USA) according to the manufacturer's order.

### Semen and testicular tissue samples collection

Cervical dislocation was used for mice physical euthanasia. Then, the left caudal part of epididymis was collected. The tissue was placed in a petri dish containing 2 cc pre-heated α-MEM medium supplemented with 15 mg/mL bovine serum albumin. The left testis was also isolated for histologic evaluation, and after weighing, it was placed in a 10% formalin solution.

### Sperm parameters evaluation 

#### Sperm motility

On a warm stage plate, the cauda part of epididymis was divided into small pieces and placed in the incubator for 30 mins at 37°C and 5% CO${}_{2}$. Then, the motility of 200 sperm under an optical microscope (Nikon, TS110) was evaluated and classified into two motile and immotile groups. Motility rate was calculated as the percentage of motile sperm (fast and slow motile) to the total evaluated sperm.

#### Sperm count

Initially, 10 μL of semen sample was well mixed with 10 μL of sperm fixation solution (formalin/sodium bicarbonate) and then 10 μL of this mixture was taken on Neubauer chamber. The sperms were counted in four large squares under an optical microscope. The mean of the total number of counted sperm was multiplied in the dilution factor and 10${}^{4}$. Results were reported as sperm count per mL (18).

#### Sperm viability and morphology

Sperm viability was evaluated by eosin-nigrosin staining. First, the semen (5 μL) was mixed with 1% eosin-10% nigrosin (5 μL) (Merck, Germany), and after 30 sec, the mixture (5 μL) was removed and a sperm smear was prepared. According to the WHO guideline (19), the sperm that is alive becomes colorless and shiny, and those that are dead becomes colored - dark red to purple. At least 200 sperm cells in each sample were evaluated randomly in five fields based on their color. The viability rate was reported as the percentage of alive sperms to the total number of counted sperms. The same slide was also used to evaluate sperm morphology. The percentage of sperm with normal morphology was calculated as the mean percentage of sperm count with normal morphology to the total assessed sperm. An abnormal morphology was determined by considering abnormalities in each of the three parts of the sperm: head, middle piece, and tail. These parameters were assessed under a light microscope (Olympus CX21, Tokyo, Japan) at a magnification of 400°.

#### Testicular morphometry and histology assessment

The left testis' diameters and weight were measured using digital caliper and digital scale, respectively. It was fixed in 10% formalin, dehydrated in 70, 90, and 100 % alcohols and embedded in paraffin. The three micron sections of paraffin blocks were taken using a microtome (Did sabz Company, Iran) and stained with hematoxylin-eosin (Merck, Germany). The number of Leydig cells in 10 fields was randomly counted under an optical microscope with a magnification of 200°.

### Ethical consideration

The present study was managed at the Kerman University of Medical Sciences, Kerman, Iran, and the experimental protocols were approved by the Animal Ethics Committee of the Kerman University of Medical Sciences, Kerman, Iran (IR.Kmu.REC.1392.588).

### Statistical analysis

The statistical Package for the Social Sciences software, version 22 (SPSS, Chicago, IL, USA) was used for statistical analysis. At first, one-sample Kolmogorov-Smirnov test was used. One-way analysis of variance (ANOVA) and then Tukey test (as a post hoc test) were used for the parametric data (sperm motility, morphology, tail abnormality, viability, Leydig cell number, and body weight). Kruskal-Wallis and Mann-Whitney U tests were used if the data had nonparametric distribution (sperm count, head and neck abnormality, testosterone hormone, testis weight, length, width). Data were expressed as mean ± standard error of the mean (SEM). P < 0.05 was considered as a significant difference.

## 3. Results

### The analysis of changes in body weight and testicles

As shown in Table I, body weight was not significantly different in control and date groups (p = 0.96). Administration of FA significantly reduced body weight (p ≤ 0.001). Although pre-treatment with DPFE could significantly reduce the weight loss induced by FA administration (p = 0.028), it still showed a significant difference compared with the control (p = 0.006) and date (p = 0.02) groups. Our data showed that receiving DPFE could significantly increase testicular weight compared to the control group (p = 0.002). The weight, length, and width of the testis in the group receiving only FA had significantly reduced compared with the control group (p ≤ 0.001). However, this weight loss in the animals that received DPFE before FA exposure was not observed compared to the control group (p = 0.07), although there was a significant difference with the date fruit-recipient group (p ≤ 0.001). In the DT + FA group, the length and width of testis did not reach to those of the control mice (Table I).

### The analysis of sperm parameters

#### Sperm viability and count

There was a significant difference in the sperm count between the DT and the control groups (p ≤ 0.001), while FA administration significantly reduced the number of sperm in comparison with the control (p ≤ 0.001) and DA (p ≤ 0.001) groups. The sperm count in the DT + FA group significantly increased in comparison to the FA group (p = 0.001); however, there was still a significant difference with the control (p ≤ 0.001) and DT groups (p ≤ 0.001) (Table II). The evaluation of sperm viability showed that i.p. administration of FA could significantly reduce the mean number of alive sperms to 57.11 ± 1.44 compared to the control (81.11 ± 1.26) and DT (79.7 ± 1.50) groups (p ≤ 0.001). Although pretreatment with date fruit extract (67.62 ± 3.77) could significantly increase the percentage of alive sperm cells compared to the FA group (p = 0.001), there was still a significant decrease compared to the control (p = 0.001) and DT groups (p ≤ 0.001) (Table II).

#### Sperm motility

The percentage of motile sperms showed a significant difference between the control and date groups (p ≤ 0.001). Receiving FA for 35 days significantly reduced sperm motility compared with the control and DT groups (p ≤ 0.001). This decrease was markedly compensated with pretreatment with DPFE compared with the FA group (p = 0.008), although it did not reach to those of the control and DT groups (p ≤ 0.001) (Table II).

#### Sperm morphology

**Figure 1:**
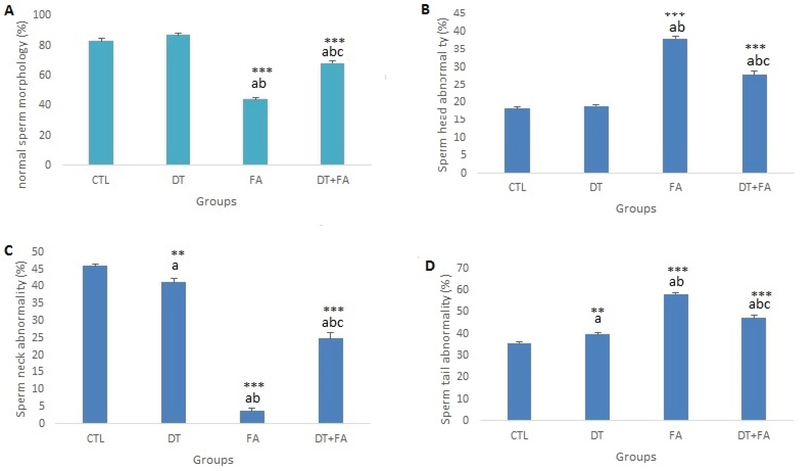
The effect of receiving date fruit extract alone or before formaldehyde administration on mice sperm morphology in different groups. ${}^{a}$Significant difference versus control group; ${}^{b}$Significant difference versus DT group; ${}^{c}$Significant difference versus FA group, DT + FA group. **p ≤ 0.01; ***p ≤ 0.001.

**Figure 2:**
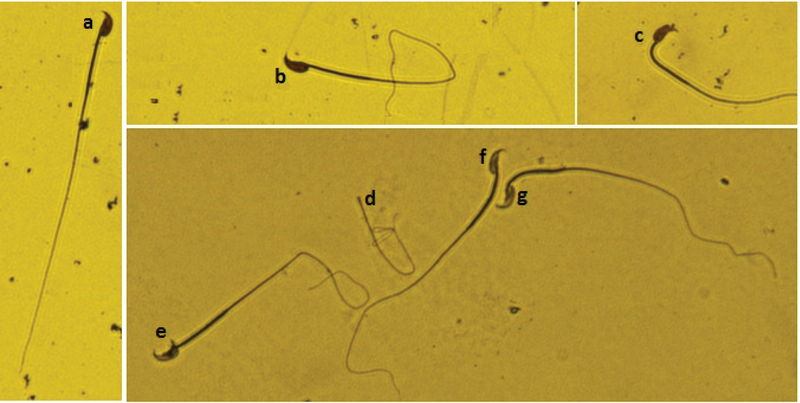
Light micrographs of mice sperm morphology (eosin/nigrosin staining, 640° total magnifications). High percent of abnormal morphology in sperm cells of mice exposed to FA was seen rather than other groups. (a) normal sperm cell; (b) bent body; (c) bent neck; (d) without head; (e) bent body and looped tail; and (f, g) bent tail.

The effects of receiving DPFE (alone), FA (alone), and pretreatment with DPFE before the administration of FA on normal morphology of sperm are depicted in Figures 1 and 2. There was no significant difference between the control and date groups in the percentage of sperm with normal morphology (p = 0.28). FA administration could significantly reduce the percentage of normal sperm morphology compared to the control and date groups (p ≤ 0.001). The percentage of sperm normal morphology in animals that received DPFE before i.p. administration of FA increased significantly compared to the FA group (p = 0.001) but still showed a significant difference with those of control (p = 0.001) and date fruit groups (p ≤ 0.001) (Figure 1A). The analysis of classification of sperm morphology anomalies also showed that FA administration significantly increased the sperm head (Figure 1B) and tail (Figure 1D) abnormalities compared to those of the control (p = 0.001) and date groups (p ≤ 0.001). In spite of the significant improvement in head and tail sperm anomalies in the pretreatment group compared to FA group (p = 0.001), they still had higher levels than those of the control and date groups (p = 0.001).

#### The analysis of serum testosterone levels and Leydig cell count

The long-term administration of FA induced a significant decrease in the testosterone level and Leydig cells count when compared to the control and DT groups. The testicular tissue in mice that received the DPFE prior to FA administration showed a significant increase in the level of this hormone and Leydig cell number when compared to the FA group. However, a significant difference in the number of Leydig cells was observed between the DT + FA group and the control and DT animals (Table III).

### The analysis of testicular tissue parameters

**Figure 3:**
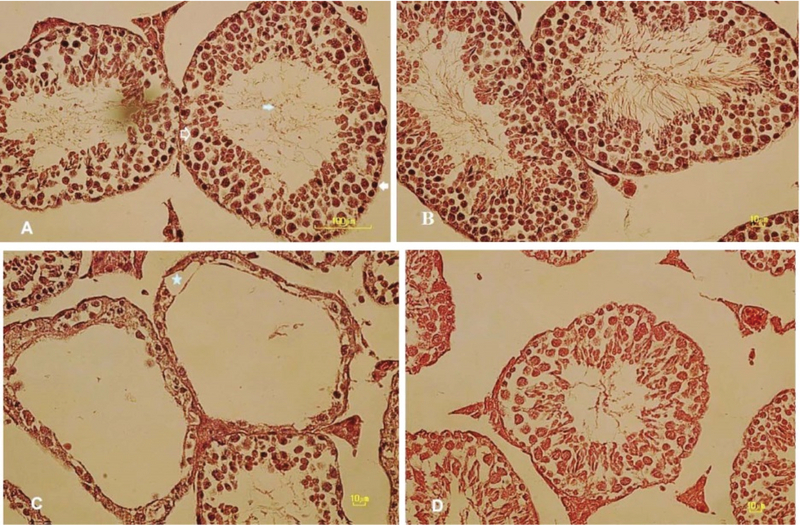
Morphology of seminiferous tubules in the testis of mature adult mice in different groups (hematoxylin and eosin staining). Control group (A), treated with date fruit extract; DT group (B), treated with FA; FA group (C) treated with date fruit extract before receiving FA; DT + FA group (D). In control (A) and DT (B) groups, seminiferous tubules were normal. Active spermatogenesis and normal arrangement of spermatogenic cell line including spermatogonia (white arrow), primary spermatocyte (without color arrow), and released sperm in lumen (blue arrow) were observed. Photomicrograph of testis in FA group (C) showed vacuolization in seminiferous epithelium (blue star) and also absence of seminiferous epithelium in many tubules. Spermatogenesis arrested in almost all seminiferous tubules so the absence of sperm are obvious in the entire lumen of tubules. Pretreatment with date fruit extract before FA administration (D) could partially ameliorate FA destructive effects on testicular tissue so that spermatogenic cell line exhibit a better order than the FA group and sperm cells observe in lumen (Olympus IX71, Japan; Magnification 400x).

As shown in Table I, animals that received FA for 35 days showed a significant decrease in the testicular length and width compared with the control group (p ≤ 0.001). Although pretreatment with date fruit significantly increased these parameters of the testis in comparison with the FA group (p ≤ 0.001 and p = 0.001, respectively), it couldn't reach to the level of control group (p ≤ 0.001 and p = 0.001, respectively). In addition, the results showed that the length of the testis increased significantly with the use of the date fruit extract compared with the control group (p = 0.02). Also, histological examination (Figure 3) showed that in the control and date groups, germinal epithelium of seminiferous tubules had a significant thickness, and active spermatogenesis was obvious (Figure 3A and B). In the testis of mice treated with FA, the thickness of germinal epithelium decreased or was totally destructed and vacuolated in many seminiferous tubules (Figure 3C). Also, irregular spermatogenic cell line was observed in some tubules so that the number of mature spermatozoa in lumen of tubules reduced markedly. Improvement of testicular tissue structure was obvious with date extract pretreatment before FA administration (Figure 3D). In comparing with the FA group, active spermatogenesis and increased released sperm in lumen was noticeable in many tubules, although different degrees of tubule destruction and germinal cell degeneration were observed in some tubules, and epithelial thickness decreased compared to the control group.

**Table 1:** The results of body weight and testis parameters in different groups


**Groups**	**Body weight (gr)**	**Length of testis (mm)**	**Width of testis (mm)**	**Testis weight (gr)**
**Control**	31.05 ± 1.06	8.15 ± 0.02	5.65 ± 0.1	0.13 ± 0.006
**DT**	30.36 ± 0.71	7.81 ± 0.31${}^{a}{}^{*}$	5.79 ± 0.06	0.17 ± 0.007${}^{a*}$
**FA**	23.16 ± 0.72${}^{(ab)***}$	5.06 ± 0.17${}^{(ab}{}^{)***}$	3.64 ± 0.06${}^{(ab)***}$	0.082 ± 0.004${}^{(ab)***}$
**DT + FA**	27 ± 0.22${}^{a}{}^{**}{}^{(}{}^{bc)}{}^{*}$	7.1 ± 0.09 ${}^{(abc}{}^{)}{}^{**}$	4.81 ± 0.04 ${}^{(abc)***}$	0.11 ± 0.005${}^{b*c}{}^{**}$
${}^{a}$Significant difference compared with control group; ${}^{b}$Significant difference compared with DT group; ${}^{c}$Significant difference compared with FA group All values are expressed as Mean ± SEM. *p ≤ 0.05; **p ≤ 0.01; ***p ≤ 0.001 DT: Date fruit extract FA: Formaldehyde DT + FA: Date fruit extract + formaldehyde				

**Table 2:** The effect of receiving date fruit extracts alone or before formaldehyde administration on sperm parameters (count, viability, and motility) of adult male mice in different experimental groups


**Groups**	**Sperm count (×10${}^{6}$ ml${}^{-1}$)**	**Sperm viability (%)**	**Sperm motility (%)**
**Control**	212 ± 1.82	81.11 ± 1.26	53.83 ± 1.68
**DT**	237.5 ± 3.04${}^{a***}$	79.7 ± 1.50	72.47 ± 1.34${}^{a***}$
**FA**	63.11 ± 0.95${}^{(ab)***}$	57.11 ± 1.44${}^{(ab)***}$	19.41 ± 3.62${}^{(ab)***}$
**DT + FA**	89.75 ± 2.01${}^{(abc)***}$	67.62 ± 3.77${}^{(ab)***c}{}^{**}$	30.73 ± 1.80${}^{(ab)***c}{}^{**}$
${}^{a}$Significant difference compared with control group; ${}^{b}$Significant difference compared with DT group; ${}^{c}$Significant difference compared with FA group All values are expressed as Mean ± SEM **p ≤ 0.01; ***p ≤ 0.001 DT: Date fruit extract FA: Formaldehyde DT + FA: Date fruit extract + formaldehyde			

**Table 3:** The effect of receiving date fruit extract alone or before formaldehyde administration on the testosterone hormone level and the number of Leydig cells of adult mice in different studied groups


**Groups**	**Testosterone concentration (ng/ml)**	**Leydig cell number**
**Control**	8.82 ± 0.09	34.55 ± 1.15
**DT**	9.57 ± 0.09${}^{a}{}^{**}{}^{*}$	35.2 ± 0.95
**FA**	7.97 ± 0.02 ${}^{(ab}{}^{)***}$	26 ± 0.7${}^{a***b**}$
**DT + FA**	8.49 ± 0.1 ${}^{\left(bc\right)}{}^{**}{}^{*}$	30.87 ± 0.64${}^{\left(ab\right)}{}^{**}{}^{*c**}$
${}^{a}$Significant difference compared with control group; ${}^{b}$Significant difference compared with DT group; ${}^{c}$Significant difference compared with FA group All values are expressed as Mean ± SEM **p ≤ 0.01; ***p ≤ 0.001 DT: Date fruit extract FA: Formaldehyde DT + FA: Date fruit extract + formaldehyde		

## 4. Discussion 

The current study showed that i.p. injection of FA had harmful effects on testicular tissue parameters (testicular diameters, destruction of seminiferous tubules, and degeneration of germinal cells) as well as sperm parameters including sperm motility, viability, count and normal morphology when compared to controls. Administration of DA for 35 days before FA administration could ameliorate these negative effects caused by FA injection to mice.

Several studies have also demonstrated that FA exposure by various dosage, duration, and methods such as oral (20) and i.p. administration (21) or inhalation (22) could negatively affect male reproductive systems; sperm count, motility, viability (23), and also testes histology, morphometry, and spermatogenesis. Our study with i.p. administration of 0.25 mg/kg FA for 35 days indicated a significant decrease in the sperm count, motility, and viability.

The mechanism of FA-induced deficiency of testicular and sperm parameters has been explained partly. It is reported that FA administration could damage testicular tissue due to oxidative stress (24). Previous studies showed a significant reduction in the level of testicular enzymatic antioxidants that are involved in scavenging of excessive ROS such as superoxide dismutase, glutathione peroxidase, catalase and reduced glutathione, as well as an increase in the level of testicular malondialdehyde; a major product of lipid peroxidation induced by excessive ROS (5, 24). An excessive production of free radicals along with reduction of antioxidant enzyme activity in testicular tissue may cause seminiferous tubules atrophy and germ cells apoptosis (25) that leads to decline the sperm count. Soleimanzadeh and co-workers reported an increased and decreased expression level of Bax (pro-apoptotic gene) and Bcl-2 (anti-apoptotic gene), respectively, in testicular tissue of FA-treated NMRI mice. They concluded that FA-induced apoptosis may reduce spermatogenenic germ cells and sperm count (5). Also, recently, Afrigan and colleagues reported a marked rise of apoptotic spermatogenic germ cells number in male rats that exposed to FA (23).

The membrane of sperm cell is rich in unsaturated fatty acids, which makes it prone to damage by free radicals. An increase in the production of free radicals following FA administration may cause lipid peroxidation of the outer membrane of the sperm cell. The lipid peroxidation of the sperm membrane can affect sperm motility by decreasing the enzymatic activity of the sodium/potassium ATP as pump which is necessary for sperm motility (26). Naghdi and colleagues suggested that FA may also have a negative effect on the main cells of epididymis and reduce the secretion of these cells that are necessary to induce maturation and sperm motility (6). Our findings confirmed the negative effect of FA i.p. administration on NMRI mice spermatogenesis and sperm parameters compared to the control animals. However, FA administration showed deleterious effects on testicular tissue and sperm cell parameters that may negatively affect male fertility potential.

Normal function of male reproductive system is dependence to the usual level of male sex hormone; testosterone. This hormone secretes from Leydig cells. Deficiency in testosterone biosynthesis and secretion leads to a deficit of spermatogenesis and sperm formation. Previous studies reported a reduction of testosterone hormone level and Leydig cells number (4) following FA exposure. They attributed these negative effects to FA-induced oxidative stress. Although the main function of FA has not been properly investigated, it has been suggested that FA generates ROS by activating enzymes responsible for the production of free radicals as well as inhibiting free radical scavenger system (5). Our data obtained in FA-treated mice in agreement with previous studies showed a significant reduction of Leydig cell number and testosterone level. Our results may be explained by a reduction in antioxidants level of testicular tissue (5) and serum (4) after long-term exposure to FA. This imbalance between testicular oxidants and antioxidants may cause Leydig cell apoptosis due to an increase in the expression of BAX protein; a pro-apoptotic factor (5) and so lead to a reduction in Leydig cell number and dysfunction and then the decreasing of testosterone hormone level.

However, date fruit extract treatment before FA i.p. administration had protective effects on sperm and testicular parameters in FA-treated animals. The primary phytochemical testes discovered the presence of fat, carbohydrate, protein, fiber, minerals (calcium, iron, fluorine, and selenium), vitamins (C, B1, B2, B3, and A), and significant phytochemicals includings flavonoids, phenols, ferulic acid, and anthocyanins (27). previous studies reported the antioxidant activity of date fruit (28, 29). This feature is attributed to the phytochemical compounds such as phenolic acids, flavonoids, anthocyanins, and mineral selenium (29). Sadeghi and colleagues demonstrated the antioxidant activity of Iranian date fruit (30). This compound acts via neutralization of superoxide and hydroxyl radicals and also inhibition of protein oxidation and peroxidation (31, 32). Previous studies reported the significant improvement effect of receiving date fruit extract as natural antioxidant therapy in testis toxicity induced by oxidative stress (9, 12, 14). They showed an increase in antioxidant enzymes activity when animals received date fruit extract along with toxin compared to animals receiving toxin alone. Also, a protective effect of date fruit extract on Leydig cells vitality is reported. Mehraban and co-workers showed that using this extract at a concentration of 120 mg/kg for 35 days, with its anti-apoptotic effect, protects the rats' Leydig cells and thus could increase the testosterone levels (33). However, the gonadotropic effects of this extract have already been reported (34). It seems that Leydig cells apoptosis and neurogenesis destroying of testicular tissue due to FA administration may ameliorate by increasing of testicular tissue antioxidant capacity following date fruit extract receiving prior to FA administration (22). In line with the reported studies, our findings showed a significant protective effect of date extract on sperm parameters and testicular changes in FA-treated animals that may be related to its high antioxidant effect. However, further molecular and antioxidant studies are needed to determine the exact machanism of Bam date fruit extract on testicular toxicity induced by FA.

## 5. Conclusion

The current study demonstrates that i.p. administration of FA had deleterious effects on testis weight, width, and length, Leydig cell number, testosterone hormone level, and also sperm parameters (motility, count, viability, and normal morphology) in NMRI mice. However, oral administration of date fruit extract prior to FA administration indicated protective effects that might be related to its antioxidant properties. Therefore, it is concluded that therapeutic plants such as date fruit with cheap price and no chemical side effects can be applied as effective protecting agent to reduce the deleterious effects of FA. However, further studies are needed to determine of their exact mechanism on testicular and sperm parameters.

##  Conflict of Interest

The authors declare that they have no competing and conflict interests.
